# Cellular ciliary phenotyping indicates pathogenicity of novel variants in *IFT140* and confirms a Mainzer–Saldino syndrome diagnosis

**DOI:** 10.1186/s13630-018-0055-2

**Published:** 2018-02-23

**Authors:** Machteld M. Oud, Brooke L. Latour, Zeineb Bakey, Stef J. Letteboer, Dorien Lugtenberg, Ka Man Wu, Elisabeth A. M. Cornelissen, Helger G. Yntema, Miriam Schmidts, Ronald Roepman, Ernie M. H. F. Bongers

**Affiliations:** 1 Department of Human Genetics (855), Radboud University Medical Centre, PO-Box 9101, 6500 HB Nijmegen, The Netherlands; 2 Radboud Institute for Molecular Life Sciences, Radboud University Medical Centre, PO-Box 9101, 6500 HB, Nijmegen, The Netherlands; 3 Department of Pediatric Nephrology, Radboud University Medical Center, Nijmegen, The Netherlands; 4 Donders Centre for Neuroscience, Radboud University Medical Center, PO-Box 9101, 6500 HB Nijmegen, The Netherlands; 5 Center for Pediatrics and Adolescent Medicine, University Hospital Freiburg, Freiburg University Medical Faculty, Freiburg, Germany

**Keywords:** Mainzer–Saldino syndrome, MZSDS, SRTD9, UREC, IFT140

## Abstract

**Background:**

Mainzer–Saldino syndrome (MZSDS) is a skeletal ciliopathy and part of the short-rib thoracic dysplasia (SRTD) group of ciliary disorders. The main characteristics of MZSDS are short limbs, mild narrow thorax, blindness, and renal failure. Thus far, variants in two genes are associated with MZSDS: *IFT140,* and *IFT172*. In this study, we describe a 1-year-old girl presenting with mild skeletal abnormalities, Leber congenital amaurosis, and bilateral hearing difficulties. For establishing an accurate diagnosis, we combined clinical, molecular, and functional analyses.

**Methods:**

We performed diagnostic whole-exome sequencing (WES) analysis to determine the genetic cause of the disease and analyzed two gene panels, containing all currently known genes in vision disorders, and in hearing impairment. Upon detection of the likely causative variants, ciliary phenotyping was performed in patient urine-derived renal epithelial cells (URECs) and rescue experiments were performed in CRISPR/Cas9-derived *Ift140* knock out cells to determine the pathogenicity of the detected variants in vitro. Cilium morphology, cilium length, and intraflagellar transport (IFT) were evaluated by immunocytochemistry.

**Results:**

Diagnostic WES revealed two novel compound heterozygous variants in *IFT140*, encoding IFT140. Thorough investigation of WES data did not reveal any variants in candidate genes associated with hearing impairment. Patient-derived URECs revealed an accumulation of IFT-B protein IFT88 at the ciliary tip in 41% of the cells indicative of impaired retrograde IFT, while this was absent in cilia from control URECs. Furthermore, transfection of CRISPR/Cas9-derived *Ift140* knock out cells with an IFT140 construct containing the patient mutation p.Tyr923Asp resulted in a significantly higher percentage of IFT88 tip accumulation than transfection with the wild-type IFT140 construct.

**Conclusions:**

By combining the clinical, genetic, and functional data from this study, we could conclude that the patient has SRTD9, also called Mainzer–Saldino syndrome, caused by variants in *IFT140*. We suggest the possibility that variants in *IFT140* may underlie hearing impairment. Moreover, we show that urine provides an excellent source to obtain patient-derived cells in a non-invasive manner to study the pathogenicity of variants detected by genetic testing.

**Electronic supplementary material:**

The online version of this article (10.1186/s13630-018-0055-2) contains supplementary material, which is available to authorized users.

## Background

Short-rib thoracic dysplasia (SRTD) refers to a group of ciliopathies characterized by skeletal abnormalities, that are classified into 18 molecular subclasses; SRTD1-16, EVC, and EVC2. The majority of genes mutated in SRTDs encode intraflagellar transport (IFT) proteins, while a small subset encode proteins involved in hedgehog signaling, ciliogenesis, or DNA damage response signaling [1–9]. IFT is required for cilium assembly, maintenance, and disassembly. Two axonemal transport modules can be distinguished: the IFT-B complex, powered by the kinesin-2 motor, for transport in anterograde direction (from ciliary base to tip), and the IFT-A complex, powered by the dynein-2 motor, for retrograde transport (from ciliary tip to base). Variants in genes encoding subunits of the IFT-A particle are associated with SRTD types 3–5, 7–9, 11, and 15 [1, 2, 4, 5, 8]. The characteristic skeletal findings of SRTDs comprise short ribs and a narrow thorax and are often combined with short stature, pelvic deformities, polydactyly, and brachydactyly. In addition to the skeletal abnormalities, other organ systems can be involved including the kidneys, the liver, and the central nervous system [1]. Since the phenotypic overlap between different SRTD subclasses is substantial, the classification is based on the underlying genetic defect and not on the clinical phenotype. Establishing an accurate diagnosis as early as possible is essential to provide the best-personalized care for the patient. For example, monitoring renal function is likely to be more important for patients carrying a pathogenic variant in *IFT140*, *TTC21B*, or *WDR19* than for patients who carry *DYNC2H1* or *IFT80* variants, as the latter two are primarily associated with a skeletal phenotype and *IFT140*, *TTC21B*, and *WDR19* are associated with a skeletal phenotype accompanied by renal insufficiency and retinal degeneration [2, 10].

The effect of variants in ciliary genes can be studied at the protein level by analyzing the ciliary phenotype in patient-derived cells. Skin biopsies are commonly used to obtain patient-derived fibroblasts; however, this is an invasive and painful procedure. In 2015, Ajzenberg et al. reported on a non-invasive method to obtain patient-derived cells from urine [11]. Urine-derived renal epithelial cells (URECs) can be used to study the ciliary phenotype and are an excellent alternative for fibroblasts. In addition, since the development of cystic kidneys is one of the hallmarks of ciliopathies, it is beneficial to study the ciliary phenotype in cells that originate from this affected organ.

In this study, we describe the use of URECs and CRISPR/Cas9 knockout cell lines to determine the pathogenicity of two novel missense variants in *IFT140* in a 1-year-old girl clinically diagnosed with a SRTD phenotype. By showing defective retrograde transport in patient-derived URECs and mutant IFT140 transfected CRISPR/Cas9-derived *Ift140* knockout cells, we confirm the clinical and molecular diagnosis of SRTD9/Mainzer–Saldino syndrome in this patient.

## Methods

### Collection of samples

Genomic DNA of patient II-3 was isolated from whole blood and used for WES. Urine was collected from the patient, her unaffected parents, and three healthy controls to study the ciliary phenotype.

### Diagnostic whole-exome sequencing

Diagnostic WES was performed as described previously [12]. In brief, we analyzed the vision disorder gene panel (version DG 2.3.2) and the hearing impairment gene panel (version DG 2.3.2) [13, 14]. Exonic and intronic positions − 20 till + 8 variants with a frequency < 5% in dbSNP, < 1% in in-house database, and < 5% in the Exome Aggregation Consortium (ExAC) were evaluated and classified based on the guidelines for variant classification determined by the Association for Clinical Genetic Science (ACGS) [15]. The same filtering strategy was used for the analysis of the complete WES dataset.

### Urine-derived renal epithelial cell isolation and culture

The collection of urine, isolation of cells, and culturing of URECs was performed as described previously [11]. In brief, midstream urine was collected and refrigerated for up to 4 h. Cells were retrieved from the urine, washed once, and cultured in 1 ml primary UREC culture medium consisting of a 1:1 ratio Dulbecco’s Modified Eagle’s medium (DMEM): Ham’s F12 Nutrient Mix containing 10% FCS, 0.1 mg/ml Primocin (ant-pm-2, Invivogen, Toulouse, France), and 1× REGM SingleQuots and growth factors (CC-4127, Lonza; Basel, Switzerland) in a 24-well plate. Cells were incubated at 37 °C with 5% CO_2_ and 0.5 ml primary UREC culture medium was added 24, 48, and 72 h after cell isolation. At 96 h after cell isolation, half of the culture medium was replaced by UREC proliferation medium consisting of REBM Basal Medium (CC-3191, Lonza; Basel, Switzerland) containing 1× REGM SingleQuots and growth factors, and 0.1 mg/ml Primocin. Every day for up to 14 days, half of the culture medium was replaced with fresh UREC proliferation medium until the URECs were visible and at approximately 80% confluence.

### Ciliary phenotyping in URECs

To determine the ciliary phenotype of the patient, healthy family members, and controls, URECs were analyzed for cilium morphology, length, and IFT. Immunocytochemistry was used to visualize the cilia as described previously [16]. In short, URECs were cultured on glass coverslips until they reached ~ 80% confluence. After fixation, permeabilization, and blocking, the coverslips were incubated with primary antibodies targeting IFT88 at 1:100 dilution (rabbit, 13967-1-AP, Proteintech Group, Manchester, UK), IFT140 at 1:100 dilution (rabbit, 17460-1-AP, Proteintech Group), acetylated-*α*-tubulin at 1:1000 (mouse, T6793, Sigma-Aldrich, Zwijndrecht, Netherlands), and RPGRIP1L at 1:500 dilution (guinea pig, SNC039, homemade [17]). Upon washing, the coverslips were incubated with fluor-labeled secondary antibodies, including goat-anti-mouse IgG Alexa Fluor 568 (1:400), goat-anti-rabbit IgG Alexa Fluor 488 (1:400), and goat-anti-guinea pig IgG Alexa Fluor 647 (1:400), obtained from Life Technologies (Bleiswijk, The Netherlands). Finally, the coverslips were embedded in Fluoromount-G (Southern Biotech, Birmingham, AL, USA) containing DAPI. Coverslips were microscopically analyzed with a Zeiss Axio Imager Z2 fluorescence microscope (Zeiss, Sliedrecht, Netherlands) equipped with an ApoTome slider. Images were obtained with ZEN 2012 software (Zeiss) and processed with Photoshop CS4 (Adobe Systems, San Jose, CA, USA) and freely available FIJI software.

### Generation of CRISPR/Cas9-derived Ift140 knockout IMCD3 cells

The CHOPCHOP website (https://chopchop.rc.fas.harvard.edu/) was used to design short-guide RNA (sgRNA) targeting exon 8 of the *Ift140* gene. The designed sgRNA-*Ift140*-E8 targets a single unique site in the mouse genome and the negative control sgRNA targets a sequence that is not present in the mouse genome. The two sgRNA sequences used are sgRNA-*ift140*-E8: ATAAAGGACGAGTAGCTATG and sgRNA-neg-ctrl: GCGAGACAGTTTGACCGTCT. Cas9 plasmid pSpCas9 (BB)-2A-GFP (PX458) containing either sgRNAs were constructed using the bbsI restriction sites. The insertion of the sgRNAs into the plasmid was verified by sequencing (data not shown).

Mouse Inner Medullary Collecting Duct 3 (mIMCD3) cells were cultured at 37 °C with 5% CO_2_ in 1:1 ratio DMEM: Ham’s F12 culture medium containing 10% FCS, 1% sodium pyruvate, and 50 µg/ml penicillin/streptomycin. mIMCD3 cells were transfected with the px458-*Ift140*-E8 or the px458-neg-ctrl construct using JetPrime according to the manufacturer’s protocol. GFP expression was checked after 24-, 48-, and 72-h post-transfection. At 72 h post-transfection, cells were harvested and prepared for FACS single-cell sorting by passing them through a 70-µm syringe Falcon (BD Bioscience). Subsequently, single-cell colony expansion occurred and genomic DNA was extracted to check for variants in *Ift140* by Sanger sequencing upon PCR amplification of the region around the targeted sequence (Additional file 1: Figure S2). Immunoblotting was performed to validate the knocking out of IFT140 on protein level. Protein lysates were made from CRISPR/Cas9 negative control cells and CRISPR/Cas9-derived *Ift140* knockout cells with or without transfection of the IFT140-Myc-DDK construct (NM_014714, cat# RC207528, OriGene, Herford, Germany). The immunoblot was stained with an antibody targeting IFT140 (IFT140 antibody was kindly provided by Dr. G. Pazour).

### IFT140 variant rescue analysis in CRISPR/Cas9-derived Ift140 knockout cells

The CRISPR/Cas9-derived *Ift140* knockout cells were no longer able to form cilia (Fig. 3b). To study the effect of the patient variants in *IFT140* on ciliogenesis and IFT88 localization, the mIMCD3 CRISPR/Cas9-derived IFT140 knockout (mIMCD3-Ift140^−/−^) cells were cultured on glass coverslips, followed by transfection with the wild-type or mutant IFT140-Myc-DDK construct (NM_014714, cat# RC207528, OriGene, Herford, Germany) using JetPrime (cat# 114, Polyplus transfection, France) according to the manufacturer’s protocol. The mutant IFT140-Myc-DDK constructs included either the p.Tyr923Asp or p.Tyr332Valfs*18 patient variants. Ciliary phenotyping analysis was performed as described in the previous section to determine the ability of the wild-type and mutant clones to rescue ciliogenesis and retrograde IFT.

## Results

### Clinical examination

**Fig. 1:**
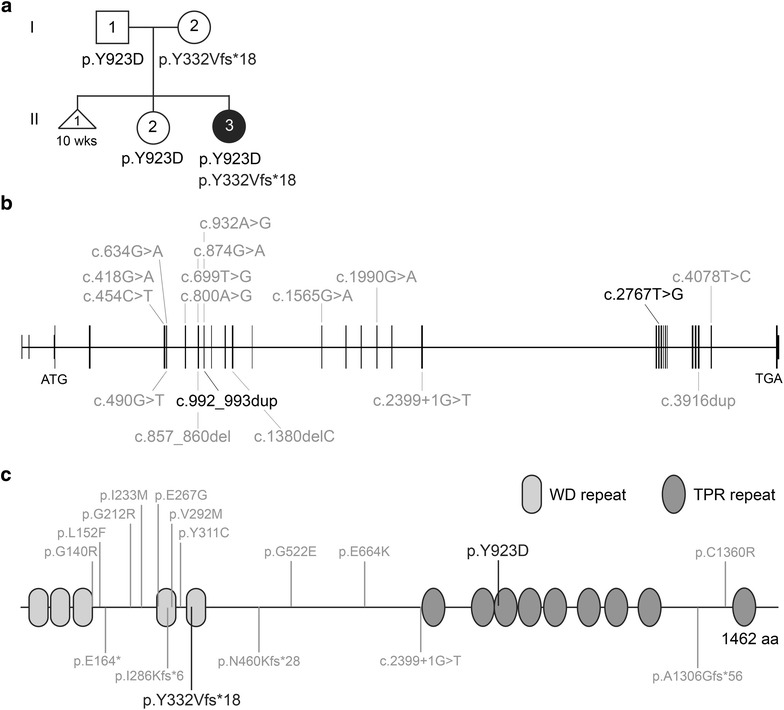
Clinical and molecular description. **a** Pedigree of the patient (II-3), including WES results for variants found in *IFT140* (NM_014714.3). **b** Schematic view of the *IFT140* gene (NM_014714.3). *IFT140* consists of 31 exons and variants, c.992_993dup and c.2767T > G, detected in patient II-3 were found in exon 9 and exon 21, respectively. The published *IFT140* variants known to cause a syndromic disease are annotated in grey. **c** Schematic view of IFT140 protein structure (NP_055529). IFT140 consists of 1462 amino acids and has five WD repeats and nine tetratricopeptide repeats (TPR). The published IFT140 variants known to cause a syndromic disease are annotated in grey. The two novel IFT140 variants are positioned in the fifth WD repeat, p.Tyr332Valfs*18, and in the third TRP repeat, p.Tyr923Asp (black)

The patient was born at term as the second child of a non-consanguineous couple from Bangladeshi descent (Fig. 1a). The first child was unaffected. The mother had previously had a miscarriage at 10-week gestation due to an unknown etiology. During the neonatal period, the patient presented with bilateral vision and hearing difficulties and was seen by a pediatrician. A behavioral observation audiometry (BOA) test, at 9 months of age, showed a bilateral moderate–severe hearing loss of a sensorineural kind necessitating hearing aids [18]. Comprehensive ophthalmologic examination at the age of 7 months diagnosed Leber congenital amaurosis (LCA). Diagnostic WES for analysis of the vision disease gene panel was requested and the patient was referred to a clinical geneticist upon retrieval of the WES results. The clinical geneticist performed a follow-up examination at 1 year of age, which showed mild skeletal abnormalities; short stature (− 1.9 SD), mild narrowing of the thorax, and brachydactyly of both hands and feet suggestive of a ciliopathy phenotype. In addition, psychomotor development was delayed; however, stimulation from the parents together with the use of prescription glasses and hearing aids contributed to marked improvement in the course of her development. There were no signs of renal insufficiency, or renal cysts on ultrasound at 12 months of age.

### Whole-exome sequencing

To determine the underlying molecular cause of the disease, a diagnostic WES analysis was performed. The vision disorder gene panel included 337 genes known to cause syndromic or non-syndromic blindness [13]. After filtering, 41 candidate variants were evaluated and only two variants were classified class 3 or higher. Both variants were found in *IFT140* (NM_014714.3) HG19:g.1637216 c.992_993dup (class 5, clearly pathogenic) and HG19:g.1575889 c.2767T > G (class 3, unknown significance) corresponding to p.Tyr332Valfs*18 and p.Tyr923Asp in IFT140 (NP_055529), respectively (Fig. 1). Segregation analysis in the parents showed bi-allelic inheritance that is in line with the expected recessive inheritance pattern of *IFT140*-associated ciliopathies. Neither of the *IFT140* variants have previously been described in the literature nor are present in the ExAC database (February 2017) [19].

To investigate the possibility of a separate genetic cause for the progressive hearing impairment present in this patient, diagnostic exome hearing impairment gene panel analysis of 132 known deafness genes was performed, but did not detect any likely pathogenic variants. Subsequently, all detected WES variants were analyzed, but no candidate pathogenic variants for hearing impairment were found. This suggests that pathogenic variants in *IFT140* could underlie the hearing impairment present in this patient.

### Ciliary phenotyping in URECs

**Fig. 2:**
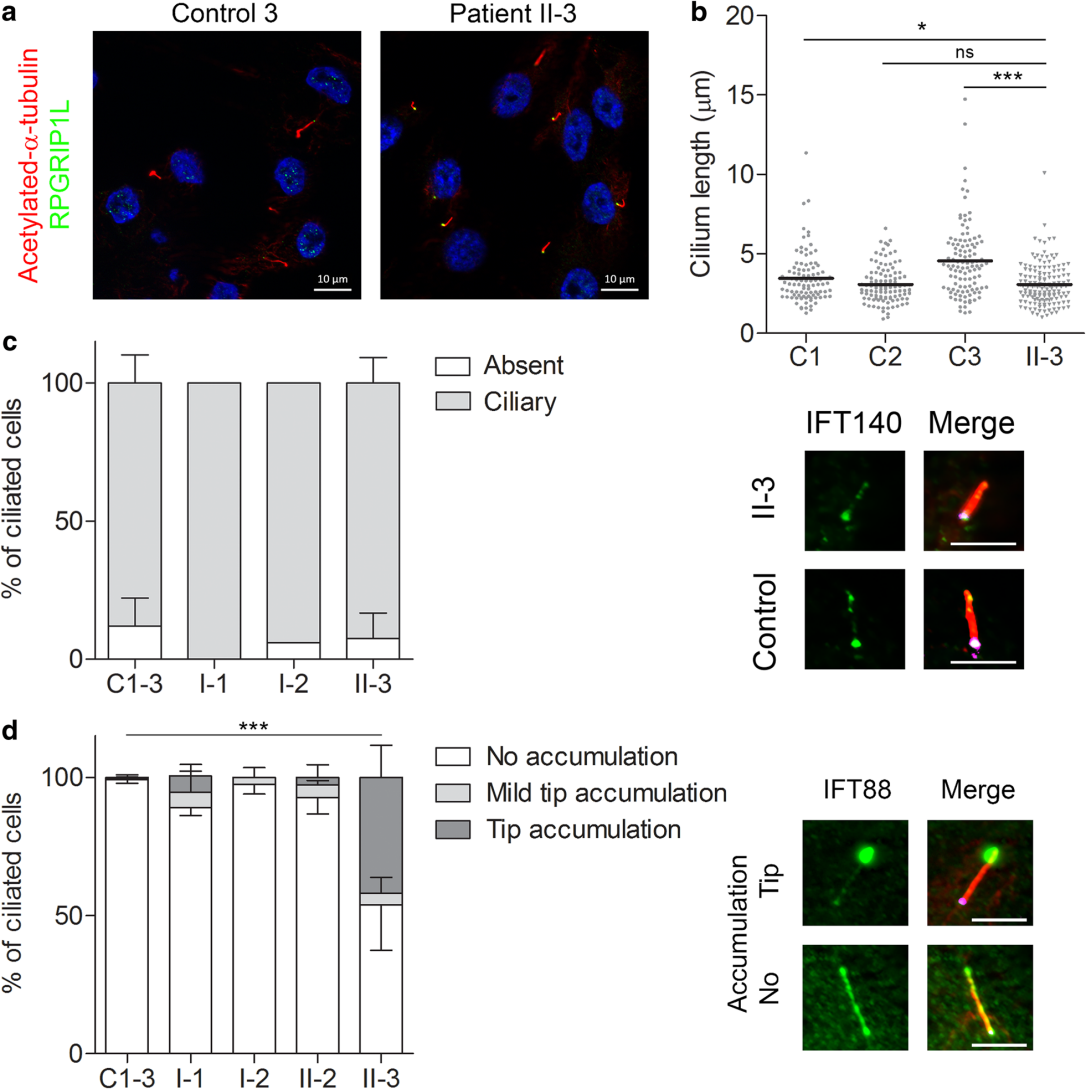
Ciliary phenotype in URECs. URECs derived from the patient (II-3) were compared to URECs from three control individuals (C1–3). **a** Representative image of URECs stained for ciliary markers, i.e., acetylated-*α*-tubulin (red) and RPGRIP1L (green). **b** Ciliary length measurements based on the acetylated-*α*-tubulin staining of the cilium. The dots represent individual cells and the horizontal line indicates the mean (N ≥ 100). Significance was measured using the unpaired two-tailed *t* test; C1 compared to C2 *p* < 0.0397; C1 compared to C3 *p* < 0.0001; C2 compared to C3 *p* < 0.0001; C1 compared to II-3 *p* < 0.0345; C2 compared to II-3 *p* < 0.9458; C3 compared to II-3 *p* < 0.0001. **c** Presence of IFT140 in the cilium. Parents (I-1 and I-2) are included in the analysis. Scale bar represents 5 µm. **d** Cilia were visualized with acetylated-*α*-tubulin (red) and RPGRIP1L (pink). The cells were analyzed for the presence of IFT88 (green) accumulation at the ciliary tip. Patient II-3 showed a significant accumulation of IFT88 in 41% of the cells (Fisher’s exact two-tailed test showed a *p* < 0.0001 when comparing the cells of the patient to those of the controls). Scale bar represents 5 µm

To determine the disruptive effect of the p.Tyr332Valfs*18 and p.Tyr923Asp variants on IFT140 protein function, we studied the ciliary phenotype in URECs of the patient and her unaffected parents, and compared them to three unrelated and unaffected controls (Fig. 2a). The ciliary length of the patient’s URECs at passage 2 (3.08 µm) was within the range measured in the controls (C1 at passage 3 = 3.47 µm, C2 at passage 2 = 3.07 µm, C3 at passage 2 = 4.30 µm) (Fig. 2b). We were able to detect IFT140 in primary cilia of the majority (90% or more) of cells from the patient, her parents, and three unrelated controls (Fig. 2c). Abnormalities were observed when studying the localization of the IFT-B protein IFT88. We detected a significant accumulation of IFT88 at the ciliary tip in 41% of the cells from the patient, and in 5% of the cells from the father as well as the healthy sibling II-2 (Fig. 2d). This accumulation was not seen in cells from the mother or in any of the unrelated controls. The measurements were carried out on URECs at passage 1–5. These data suggest that even though the mutated IFT140 localizes to the primary cilium and does not cause ciliogenesis defects, it does lead to defective retrograde transport.

### IFT140 variant rescue analysis in CRISPR/Cas9-derived Ift140 knockout cells

**Fig. 3:**
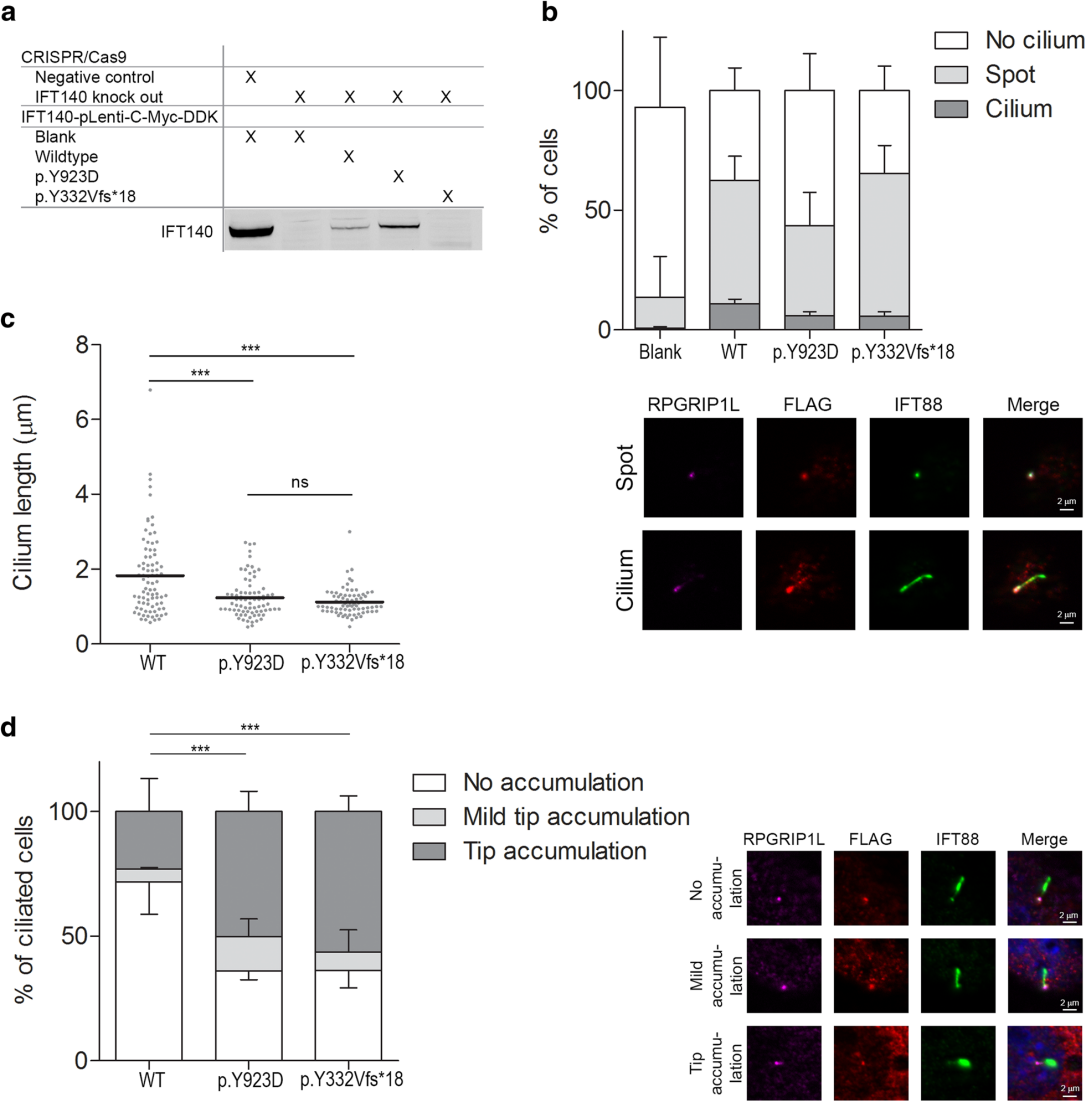
IFT140 variant rescue analysis in mIMCD3-*Ift140*^−/−^ cells. CRISPR/Cas9-derived *Ift140* knockout mIMCD3 (mIMCD3-*Ift140*^−/−^) cells were transfected with wild-type, p.Tyr923Asp or p.Tyr332Valfs*18 mutant IFT140-Myc-DDK constructs and the cilium rescue abilities were assessed. Immunofluorescent images are representative for the measurements showing the base of the cilium using RPGRIP1L (pink), an antibody targeting FLAG to visualize the IFT140-Myc-DDK construct (red), and IFT88 in green. Scale bar represents 2 µm. **a** Immunoblot analysis of IFT140 protein levels in CRISPR/Cas9 negative control mIMCD3 cells and CRISPR/Cas9-derived *Ift140* knockout mIMCD3 cells in the absence or presence of the IFT140-Myc-DDK construct. The immunoblot was stained with an antibody targeting IFT140. **b** Ciliogenesis measurements in untreated mIMCD3-Ift140^−/−^ cells and after transfection with wild-type or mutant IFT140-Myc-DDK constructs. Untreated mIMCD3-Ift140^−/−^ cells showed < 1% ciliogenesis. Transfection with the wild-type IFT140 construct rescued ciliogenesis in 11% of the analyzed cells, while both mutant IFT140 constructs, p.Tyr923Asp and p.Tyr332Valfs*18, rescued ciliogenesis in 6% of the cells (*N* = 3 with > 250 cells analyzed per experiment). **c** Length measurements of the rescued cilia based on the IFT88 staining of the cilium. The dots represent individual cells and the horizontal line indicates the mean (*N* ≥ 75). Significance was measured using the unpaired two-tailed *t* test; wild-type compared to either mutant showed a *p* < 0.0001. **d** Ciliary localization of the IFT-B protein IFT88. There is a significant increase of cilia displaying a ciliary tip accumulation of IFT88 in both the p.Tyr923Asp (50%) and the p.Y332Vfs*18 (56%) mutant IFT140 constructs compared to wild-type (23%) (Fisher’s exact two-tailed test showed a *p* < 0.0001 when comparing either mutant to wild-type IFT140). *N* = 3 with > 50–100 cells per experiment

The protein lysate of mIMCD3-Ift140^−/−^ cells validated the absence of IFT140 protein in these cells (Fig. 3a). Upon transfection with wild-type or p.Tyr923Asp mutant IFT140, a partial rescue of the IFT140 protein level was detected. There was no IFT140 signal detected in the protein lysate of mIMCD3-Ift140^−/−^ transfected with p.Tyr332Valfs*18. To exclude that the observed ciliary tip accumulation of IFT88 in patient-derived URECs was due to other variants, we assessed the ability of patient mutant IFT140 constructs to rescue ciliogenesis and determined the localization of IFT88. Ciliogenesis was restored in 11% of the analyzed cells upon rescue with wild-type IFT140. Both mutants, p.Tyr923Asp and p.Tyr332Valfs*18, rescued ciliogenesis in 6% of the cells (Fig. 3b). Defects in retrograde IFT were visualized by ciliary tip accumulation of IFT-B protein IFT88. Cilia rescued with the p.Tyr923Asp mutant IFT140 showed IFT88 ciliary tip accumulation in 50%, which is significantly more than seen in ciliated cells rescued with wild-type IFT140 (23% tip accumulation). A similar phenotype was seen in ciliated cells transfected with p.Tyr332Valfs*18 IFT140 (56% tip accumulation).

## Discussion

In this study, we used diagnostic WES to detect variants in *IFT140* as a likely cause of a clinical ciliopathy phenotype of the patient and performed cellular ciliary phenotyping to investigate the pathogenicity of these variants. The classical syndromic phenotype associated with variants in *IFT140* includes skeletal abnormalities, renal disease, and retinal dystrophy. Hearing impairment has been described only once before to be part of the *IFT140*-related ciliopathy spectrum [20], when a patient with otherwise non-syndromic retinal dystrophy, carrying variants in *IFT140,* was described with progressive hearing loss. Our patient presented with progressive hearing loss in the neonatal period, and, since the majority of congenital hearing loss has a genetic cause, we thoroughly investigated the WES data but could not detect any likely pathogenic variants in genes associated with hearing impairment. Although we cannot exclude non-genetic causes to underlie the hearing impairment of our patient, it is possible that the detected pathogenic *IFT140* variants are causative for this feature, not previously described in Mainzer–Saldino patients.

Since pathogenic variants in other IFT-A proteins could give rise to a similar cellular phenotype, we carefully checked the remaining IFT-A and dynein motor complex proteins and could not find any pathogenic variants or large homozygous copy number variations (CNVs) [21, 22]. In addition, we carefully checked *IFT172,* as this gene is known to give rise to a similar clinical phenotype [3], but did not find any (likely) pathogenic changes in this gene either. We next evaluated the detected *IFT140* variants (workflow presented in Additional file 1: Figure S1). IFT140 is part of the IFT-A core subcomplex together with IFT122 and WDR19, which combined with the peripheral subcomplex, that includes IFT43, WDR35, and TTC21B, forms the functional IFT-A complex [23, 24]. The previous studies have shown that defective retrograde transport causes an accumulation of proteins at the ciliary tip, and may result in shorter cilia [21, 22, 25, 26]. We studied URECs from the patient to determine whether the novel *IFT140* variants caused defective retrograde IFT (workflow presented in Additional file 1: Figure S1). We detected defective retrograde IFT in 41% of the cilia of the patient’s cells, while this was not seen in controls and only in a small percentage of cilia from the unaffected sibling and parents. Significant effects on ciliary length were not observed. To exclude that the observed retrograde IFT defect is caused by variants other than the ones found in *IFT140*, we additionally performed rescue experiments using CRISPR/Cas9-derived *Ift140* knockout cells. A significantly higher percentage of cilia showed IFT88 tip accumulation in *Ift140* knockout cells transfected with an IFT140 construct carrying the patient p.Tyr923Asp or the p.Tyr332Valfs*18 variants compared to wild-type IFT140.

Recent publications have shown that the IFT-A complex is not only required for retrograde transport, but that it is also essential for the ciliary entry of transmembrane proteins, including G protein-coupled receptors (GPCRs), important for the transduction of intercellular signals [27, 28]. Cells defective for TTC21B, part of the peripheral subcomplex, showed an accumulation of both IFT-A and B at the ciliary tip, and a ciliary enrichment of GPCRs. Interestingly, cells defective in IFT-A core component WDR19 showed an accumulation of IFT-B proteins at the ciliary tip, while IFT-A components and GPCRs were absent from the cilium [28]. This means that disruption of the IFT-A core subcomplex abolishes IFT-A complex formation and its entry into the cilium, not only resulting in defective retrograde IFT, but also withholding ciliary entry of GPCRs. Our data provide evidence that the defective retrograde IFT seen in the patient is caused by the mutated IFT140, though the possible effects on the ciliary entry of GPCRs were not assessed in this study.

Combining the results of clinical, molecular, and functional studies, we conclude that the patient carries two pathogenic variants in *IFT140* underlying SRTD9 also called Mainzer–Saldino syndrome. We suggest that variants in *IFT140* may cause progressive hearing impairment, as well, and that this could be part of the MZSDS phenotype. The classic MZSDS phenotype includes renal failure, which is not seen in the patient until 1.5 years of age. Therefore, monitoring of renal function of the patient needs to be continued in the future as this may decline over time.

## Conclusion

In this study, two novel *IFT140* variants were detected by diagnostic WES, suggestive for MZSDS, in a 1-year-old girl with skeletal, retinal, and hearing abnormalities. We used a non-invasive technique to obtain patient-derived cells to study the pathogenicity of the *IFT140* variants in vitro. URECs from the patient displayed defective retrograde IFT that is most likely caused by the detected *IFT140* variants. Rescue experiments with p.Tyr923Asp or p.Tyr332Valfs*18 mutant IFT140 constructs in CRISPR/Cas9-derived *Ift140* knock out cells also showed retrograde IFT defects, confirming the pathogenicity of the variants. URECs provide a great source to assess the pathogenicity of nonsense, missense, and splice site variants found with genetic testing. The combination of a thorough clinical examination, genetic testing, and functional studies established a definitive diagnosis of SRTD9.

## Additional file

**Additional file 1: Figure S1.** Schematic overview of the workflow described in this study. **Figure S2.** Sequence validation of CRISPR/Cas9-derived *Ift140* knockout cells.

## Abbreviations

ACGS: Association for Clinical Genetic Science
CRISPR/Cas9: Clustered Regularly Interspaced Short Palindromic Repeats/Cas9
EVC: Ellis-van Creveld
ExAC: Exome Aggregation Consortium (http://exac.broadinstitute.org/)
GPCRs: G-protein-coupled receptors
IFT: intraflagellar transport
MZSDS: Mainzer–Saldino syndrome
SRTD: short-rib thoracic dysplasia
URECs: urine-derived renal epithelial cells
WES: whole-exome sequencing
