# Supplementary material for: Cellular ciliary phenotyping indicates pathogenicity of novel variants in IFT140 and confirms a Mainzer–Saldino syndrome diagnosis
Source: Cilia. 2018 Feb 23;7:1. doi: 10.1186/s13630-018-0055-2 (PMC6247778; doi:10.1186/s13630-018-0055-2)
Supplement: Supplementary file 1 — Additional file 1: Figure S1. Schematic overview of the workflow described in this study. Figure S2. Sequence validation of CRISPR/Cas9-derived Ift140 knockout cells. [file 13630_2018_55_MOESM1_ESM.docx]

**Additional data**


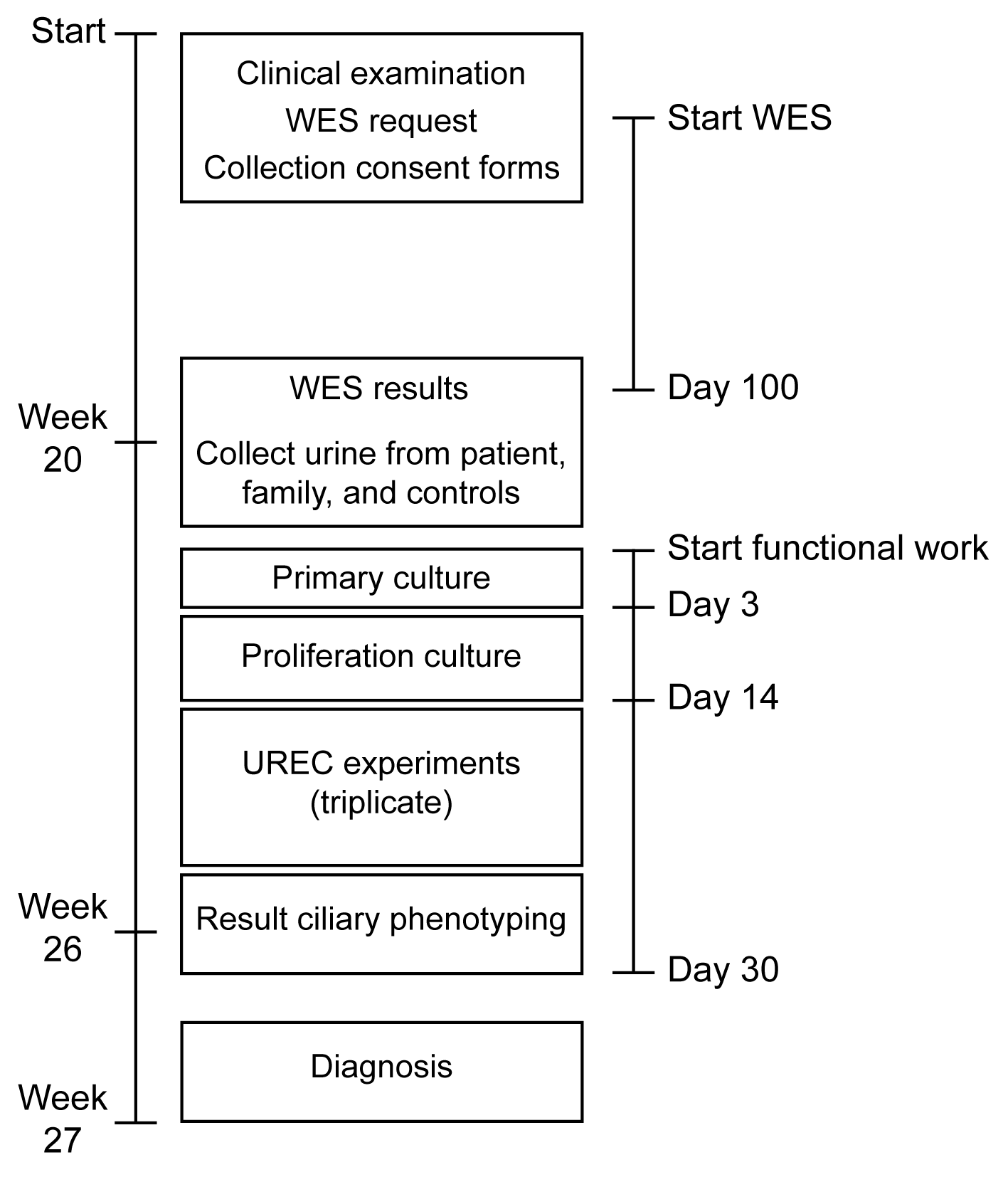


**Figure S1. Schematic overview of the workflow described in this study.**

**Sequence validation of CRISPR/Cas9-derived *Ift140* knock out cells**

gDNA from CRISPR/Cas9 treated mIMCD3 cells was extracted using DNeasy Blood & Tissue Kit (Qiagen) according to manufacturer’s instruction. The region around the targeted sequence was amplified by using the following primers: Ift140-E8, forward- TATGGGTCATGTTTGTGCAAC, reverse- CTGACCTACACTGAGTAACTGATA. PCR product was purified using ExoSAP (Thermofisher), subsequently the purified products were sent for Sanger sequencing (Figure S2).


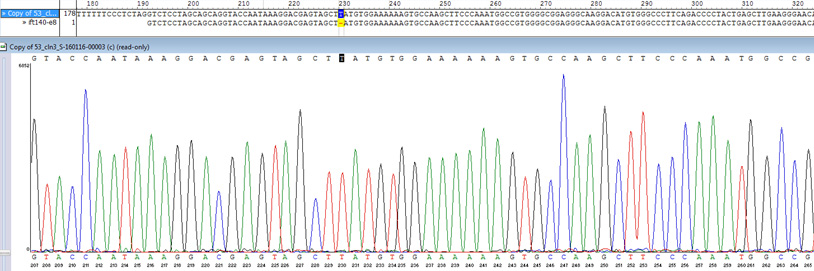


**Figure S2**. Sequence fragment of *Ift140* exon 8 containing the CRISPR/Cas9 induced mutation in the CRISPR/Cas9-derived *Ift140* knock out mIMCD3 cells.
